# Long Noncoding RNA MALAT1 Interacts with miR-124-3p to Modulate Osteosarcoma Progression by Targeting SphK1

**DOI:** 10.1155/2021/8390165

**Published:** 2021-07-29

**Authors:** Bin Liu, Xinli Zhan, Chong Liu

**Affiliations:** ^1^Department of Spine Osteopathic Surgery, The First Affiliated Hospital of Guangxi Medical University, Nanning 530022, Guangxi, China; ^2^Department of Spine Surgery, Hunan Provincial People's Hospital (The First Affiliated Hospital of Hunan Normal University), Changsha 410005, Hunan, China

## Abstract

**Introduction:**

Long noncoding RNAs (lncRNAs) have been implicated in a variety of biological functions, including tumor proliferation, apoptosis, progression, and metastasis. lncRNA metastasis-associated lung adenocarcinoma transcript 1 (MALAT1) is overexpressed in various cancers, as well as osteosarcoma (OS); however, its underlying mechanism in OS is poorly understood. This investigation aims to elucidate the mechanisms of MALAT1 in OS proliferation and migration and to provide theoretical grounding for further targeted therapy in OS.

**Methods:**

In the present study, we applied qRT-PCR to assess the MALAT1 expression in OS tissues and cell lines. The effects of MALAT1 and miR-124-3p on OS cell proliferation and migration were studied by CCK-8 and scratch assays. Cell cycle and apoptosis were tested using a flow cytometer. The competing relationship between MALAT1 and miR-124-3p was confirmed by dual-luciferase reporter assay.

**Results:**

MALAT1 was overexpressed in OS cell lines and tissue specimens, and knockdown of MALAT1 significantly inhibited cell proliferation and migration and increased cell apoptosis and the percentage of G0/G1 phase. Furthermore, MALAT1 could directly bind to miR-124-3p and inhibit miR-124-3p expression. Moreover, MALAT1 overexpression significantly relieved the inhibition on OS cell proliferation mediated by miR-124-3p overexpression, which involved the derepression of sphingosine kinase 1 (SphK1).

**Conclusions:**

We propose that lncRNA MALAT1 interacts with miR-124-3p to modulate OS progression by targeting SphK1. Hence, we identified a novel MALAT1/miR-124-3p/SphK1 signaling pathway in the regulation of OS biological behaviors.

## 1. Introduction

Osteosarcoma (OS) is a common primary bone tumor with predilection in children and adolescents, the incidence of which has been ranked as the highest of all primary malignant bone tumor types [1]. OS is characterized by high degree of malignancy and early metastasis. Many patients with OS already have advanced disease with distant metastases at the time of initial presentation, and thus it poses great challenges to clinical practitioners. OS has a dismal prognosis after metastasis has occurred although the 5-year survival of treated OS patients has significantly increased in the past decades. Recent breakthroughs in the use of targeted therapies in the management of malignant tumors, such as leukemia and lung cancer, bring beneficial inspiration for OS treatment. Thus, it is essential to explore the molecular mechanisms underlying OS tumorigenesis and progression and to identify clinically relevant biomarkers and targets for OS.

Although 93% of human genome can be transcribed into RNAs, only 2% of these RNAs can be translated to proteins. The rest of 98% of the RNAs are noncoding RNA (ncRNA) with limited or no protein-coding capacity. Among them, long noncoding RNAs (lncRNAs) are a class of RNA molecules with lengths in the range of 200–100,000 nucleotides and engaged in diverse biological processes. Increasing evidence has suggested that lncRNAs can participate in gene expression, including epigenetic regulation, transcription regulation, and posttranscriptional regulation, thus playing a pivotal role in cancer development and progression. Previous studies show that metastasis associated lung adenocarcinoma transcript 1 (MALAT1) is related to the occurrence, development, metastasis, and prognosis of multiple tumor types, including OS [2]. MALAT1 is highly expressed in OS primary tissues and cell lines, and downregulation of MALAT1 decreases proliferation, migration, invasion, and epithelial-mesenchymal transition (EMT) in OS cells. In addition, inhibition of MALAT1 can lead to cell cycle arrest and apoptosis [3–5]. However, the molecular mechanism underlying MALAT1 regulation on OS is not clear enough.

MicroRNAs (miRNAs) are a class of endogenous noncoding single-stranded RNA molecules with lengths in the range of 18–24 nucleotides. They can degrade mRNAs or inhibit mRNAs translation by binding to the 3′-untranslated regions (3′-UTR) of the target mRNA, resulting in downregulation of target gene expression. In the latest years, increasing attention has now been paid to the role of miRNAs in tumor initiation and progression [6]. Previous studies indicate that miR-124-3p is a tumor suppressor miR due to its low expression in a variety of cancers and that it may inhibit proliferation, migration, and invasion of cancer cells by suppressing different targets [7, 8]. However, the specific mechanism of miR-124-3p in OS is still obscure.

It has been reported that MALAT1 can competitively bind with miRNAs, thus indirectly regulating miRNA-target expression. This competitive binding to miRNAs is also called miRNA sponges [9, 10]. In the present study, we identified the overexpression of MALAT1 in OS and its oncogenic role in OS development. Moreover, our research validated that MALAT1 could bind to miR-124-3p, thereby competing directly with sphingosine kinase 1 (SphK1) as endogenous molecular sponges. This study identified the MALAT1/miR-124-3p/SphK1 pathway in human OS for the first time.

## 2. Materials and Methods

### 2.1. Specimen Collection

Fresh tumor specimens were surgically isolated from OS patients who were treated in Hunan Provincial People's Hospital. Adjacent healthy tissues were also taken from these patients with OS to serve as control tissue. All specimens were pathologically confirmed as OS. All of the specimens were immediately snap-frozen in liquid nitrogen and stored at −80°C until use. All study procedures conformed to the ethical standards of the Declaration of Helsinki. Approval for the study was obtained from the hospital ethics committee (approval number 2019-S14), and informed consent was obtained from all individuals.

### 2.2. Cell Source and Culturation

Human osteoblast cell line (HfoB1.19) and human OS cell lines (MG63, U2OS, and Saos-2) were all purchased from Procell Life Science & Technology Co., Ltd. (Wuhan, China).

Cell culture: HfoB1.19 cells, MG63, U2OS, and Saos-2 OS cells were cultured in DMEM medium supplemented with 10% fetal bovine serum (FBS). The media were purchased from Procell Life Science & Technology Corporation (Wuhan, China), and the FBS were purchased from Hyclone (South Logan, UT, USA). The medium contained penicillin (100 U/mL) and streptomycin (100 U/mL). All cell lines were grown in a 37°C incubator with 5% CO_2_.

### 2.3. Binding Site Prediction for MALAT1, miR-124-3p, and SphK1

Binding sites between MALAT1 and miR-124-3p were predicted with online prediction software starBase V2 (http://starbase.sysu.edu.cn/starbase2/), while binding sites between miR-124-3p and SphK1 were predicted with online prediction software TargetScan (http://www.targetscan.org/vert_72/).

### 2.4. RNA Extraction and Quantitative Real-Time Polymerase Chain Reaction (qRT-PCR)

Total RNA extraction was performed using MiniBEST Universal RNA Extraction Kit (Cat.#9767) (Takara, Dalian, China) according to the manufacturer's instructions. An amount of 1.5288 *μ*g of RNA was reverse transcribed into cDNA by using a Reverse Transcriptase kit (Primescript RT reagent kit with gDNA Eraser perfect real time). Standard qRT-PCR reactions were performed on the ABI 12K Real-Time PCR System instrument, and mRNA levels were quantified using a SYBR-Green Mix Kit (LightCycler 480 SYBER Green I Master, Roche).

miRNA extraction was performed using miRNeasy Micro Kit (QIAGEN, Valencia, CA, USA) according to the manufacturer's instructions. RNA was reverse transcribed into cDNA and then subjected to a qRT-PCR assay.

All primers were purchased from Jima Pharmaceutical Company (Shanghai, China), and all primer sequences are available in Table 1. Relative expression levels were calculated as ratios normalized against the endogenous control (GAPDH or U6 snRNA). The relative fold changes of candidate genes were analyzed using the 2^−ΔΔ^CT method.

### 2.5. Cell Transfection

Three small interfering RNA (siRNA) targeting MALAT1 sequences were designed, and the sequence with the best suppressive effect was selected and used in further studies to minimize off-target effects. Lipofectamine RNAiMAX Reagent (Life Technologies, Carlsbad, CA) was used for the transfection of various siRNA constructs into OS cells, and for luciferase reporter assay, Lipofectamine 2000 Reagent was used for the cotransfection of pmirGLO-MALAT1/SphK1-WT or pmirGLO-MALAT1/SphK1-MUT and miR-124-3p mimic or mimic-NC into HEK 293T cells. siRNA targeting MALAT1 (si-MALAT1), siRNA targeting SphK1 (si-SphK1), scrambled negative control (si-NC), miR-124-3p, miR-124-3p mimic, and NC mimics were all purchased from Jima Pharmaceutical Company (Shanghai, China).

### 2.6. Cell Counting Kit-8 (CCK-8) Assay

A CCK-8 assay was used to detect cell proliferation. 24 h after transfection, cells in the logarithmic growth phase were seeded in 96-well plates, with 5000 cells per well, and three replicates were set in each group. Cells were cultured at 5% CO2, 37°C in an incubator. After an additional 4 h incubation with 10 *μ*L CCK-8 reagent, the optical density (OD) at the 450 nm wavelength (OD450) was measured using an EnSpire Multimode Plate Reader (PerkinElmer, Woodbridge, ON, Canada) at 1, 2, 3, and 4 days after transfection.

### 2.7. Scratch Assay

A scratch assay was applied to evaluate the migration of human osteoblast cell line and OS cell lines. Two parallel lines were drawn on back of the 6-well plates with a marker pen before cell seeding, and the cells were seeded in 6-well plate after digesting. We used a 10 *μ*l pipette tip to gently draw lines on the plate when cells covered the bottom of the plate, and the width of each scratch should be as close to identical as possible. After rinsing the plate with PBS buffer for three times to remove cell debris produced by the scratching, the cells were photographed (0 h). Next, pictures were taken at 6 h, 24 h, and 48 h incubation, respectively. Finally, the pictures were collected for analysis.

### 2.8. Cell Cycle and Apoptosis Assay

Cell cycle phase distribution was measured and analyzed with CytoFLEX flow cytometer (Beckman Coulter). Cells were transiently transfected with siRNA after overnight incubation, and OS cells were collected at 48 h after transfection and washed with PBS. Then, the collected cells were fixed by 70% ethanol overnight at 4°C. Finally, DNA dye liquor was added for flow cytometry detection after ethanol removing and PBS washing. Data were collected and analyzed with the CytExpert v.2.3 software (Beckman Coulter).

Cell apoptosis was analyzed using the Annexin V-PI apoptosis detection kit (A211, Vazyme, Nanjing, China). The cells were transfected with a specific siRNA (6 × 10^4^ cells per well in a 24-well plate). The transfected OS cells were harvested and washed with PBS. Then, cells were resuspended in 100 *μ*l of Annexin Binding Buffer and incubated with 5 *μ*l of Annexin FITC and 5 *μ*l of PI for 15 min. The solutions were protected from light and incubated at room temperature. Finally, we examined cell apoptosis after adding 150 *μ*l Annexin Binding Buffer.

### 2.9. Dual-Luciferase Reporter Assay

The luciferase assays were carried out using Dual-Luciferase Reporter Assay System (Promega, Madison, WI, USA). Cells were collected and lysed for luciferase detection according to the manufacturer's instructions at 48 h after cotransfection.

### 2.10. Western Blotting

Cells were lysed using RIPA buffer (Beyotime Biotechnology, Shanghai, China). Following lysis, cells were mixed with 5 × SDS loading buffer and boiled for 5 min at 100°C. The proteins were separated by 10% SDS-PAGE and transferred onto PVDF membranes. Then, membranes were blocked with 5% milk/TBST for 1 h and subsequently incubated with primary antibodies at 4°C overnight, and secondary antibodies were diluted in 5% milk/TBST at room temperature for 1 h. The protein expressions were analyzed using an enhanced chemiluminescence (ECL) reagent and ChemiDoc™ XRS + imaging system System (Bio-Rad, CA, USA), and GAPDH served as internal reference.

### 2.11. Statistical Analysis

All statistical analyses were performed with SPSS software (version 22.0). Values are presented as the mean ± SD, and each experiment was repeated at least three times. The Fisher analysis, independent sample *t*-test, and one-way analysis of variance (ANOVA) were used as appropriate. *p* values less than 0.05 were considered statistically significant. All graphs were prepared using GraphPad Prism 7.0 software (GraphPad Software, San Diego, USA) and Adobe Illustrator (Adobe, San Jose, CA).

## 3. Results

### 3.1. LncRNA MALAT1 Plays an Important Role in Progression of OS

**Figure 1:**
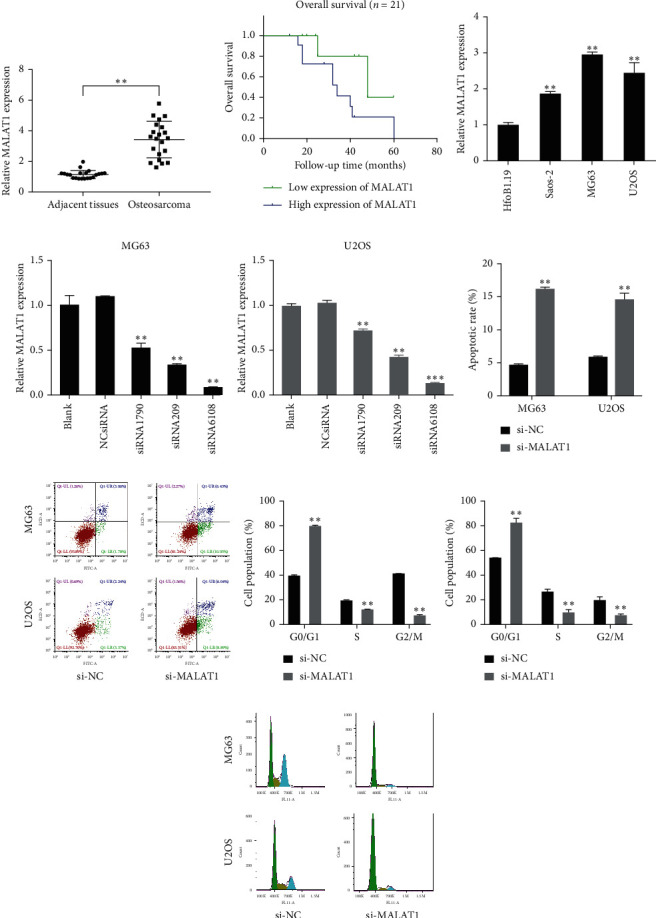
(a) Long noncoding RNA metastasis associated lung adenocarcinoma transcript 1 (MALAT1) was upregulated in osteosarcoma (OS) tissues. (b) Survival curve revealed that high MALAT1 expression was associated with a poorer overall survival in OS patients. (c) MALAT1 was upregulated in OS cell lines. The three OS cell lines Saos-2, MG63, and U2OS all had a higher level of MALAT1 expression than the normal osteoblast cell line HfoB1.19. (d, e) MALAT1 knockdown was achieved by MALAT1 small interfering RNA (si-MALAT1), especially in siRNA6108 group. (f, g) Knockdown of MALAT1 increased ratios of apoptotic cells, compared with the si-NC (negative control) group. (h–j) MALAT1 silence increased the percentage of OS cell lines in G0/G1 phase, compared to the si-NC group in MG63 and U2OS cells. ^*∗*^*p* < 0.01.

The effect of lncRNA MALAT1 on OS development and progression was investigated. The qRT-PCR results showed that MALAT1 was elevated in human OS specimens compared to adjacent healthy tissues (*p* < 0.01) (Figure 1(a)). We further explored the association between MALAT1 expression and clinical pathologic parameters. Based on the MALAT1 expression median value, OS patients were divided into two groups: high and low MALAT1 expression groups. Our data suggested that high MALAT1 expression was correlated with advanced clinical stage and distant metastasis (Table 2; *p* < 0.05). Moreover, the analysis of the survival curve revealed that high MALAT1 expression was associated with poorer overall survival in OS patients (Figure 1(b); *p* < 0.05).

Next, we measured MALAT1 expression in different OS cell lines (MG63, U2OS, and Saos-2) and human osteoblast cell line (HfoB1.19) using qRT-PCR. The results showed that MALAT1 was significantly upregulated in all the OS cell lines, particularly in MG63 and U2OS (*p* < 0.05) (Figure 1(c)). Therefore, MG63 and U2OS cell lines were selected for subsequent experimentations. Together, it was suggested that MALAT1 may significantly associated with OS development and progression.

To ensure that OS cells were effectively and specifically blocked, cells were transfected with either a control scrambled siRNA (NC-siRNA) or the MALAT1-specific siRNA (siRNA1790, siRNA209, and siRNA6108). Compared with the NC-siRNA group, MALAT1 expression was decreased in siRNA groups, especially in siRNA6108 group (Figures 1(d) and 1(e)). Thus, siRNA6108 was selected for the following experiments.

**Figure 2:**
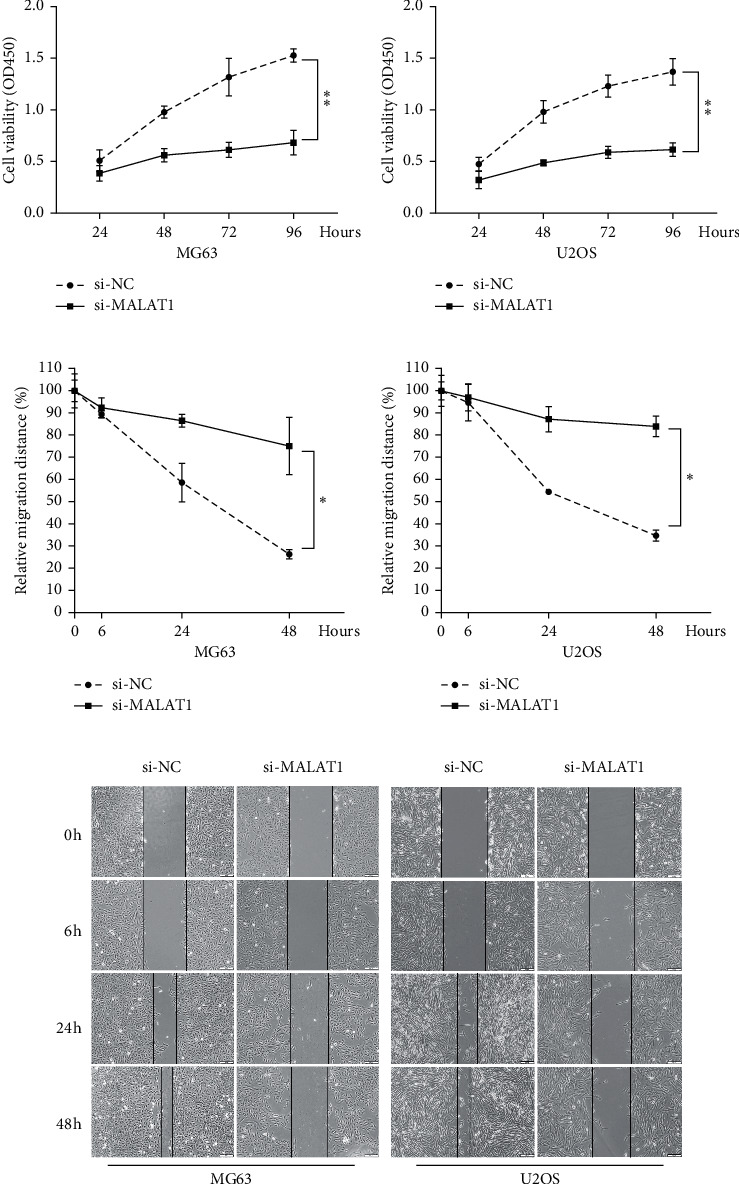
MALAT1 is associated with OS cells proliferation and migration. (a, b) CCK-8 assay showed that knockdown of MALAT1 decreased the cell viability of both MG63 and U2OS cells for up to 4 days, compared with the si-NC group. (c–e) Cell migration in MG63 and U2OS cells transfected with si-MALAT1 or si-NC was detected by scratch assay and is shown both pictorially and graphically. Compared to the si-NC group, knockdown of MALAT1 reduced the relative migration distance in both MG63 and U2OS cells for up to 48 hours. ^*∗*^*p* < 0.05 and ^*∗∗*^*p* < 0.01.

To explore the association of MALAT1 expression with OS cell cycle and apoptosis, MALAT1 siRNA (si-MALAT1) and negative control (si-NC) were transfected into two OS cell lines: MG63 and U2OS. Compared with the si-NC group, ratios of apoptotic cells were increased in cells transfected with si-MALAT1 as measured by flow cytometry analysis (Figures 1(f) and 1(g)), while MALAT1 silence increased the percentage of OS cell lines in G0/G1 phase (Figures 1(h)–1(j)). Cell proliferation and migration were determined by CCK-8 and scratch assays, respectively. When compared with the si-NC group, knockdown of MALAT1 relatively decreased the cell viability of both MG63 and U2OS cells for up to 4 days (Figures 2(a) and 2(b)). Knockdown of MALAT1 also reduced the relative migration distance in both MG63 and U2OS cells (Figures 2(c)–2(e)). Together, the above data indicated that si-MALAT1 could extremely downregulate expression level of MALAT1 and that MALAT1 decreases the percentage of G0/G1 phase, inhibits apoptosis, and promotes cell proliferation and migration in human OS cells.

### 3.2. Negative Regulation Relationship between miR-124-3p and MALAT1 in OS

**Figure 3:**
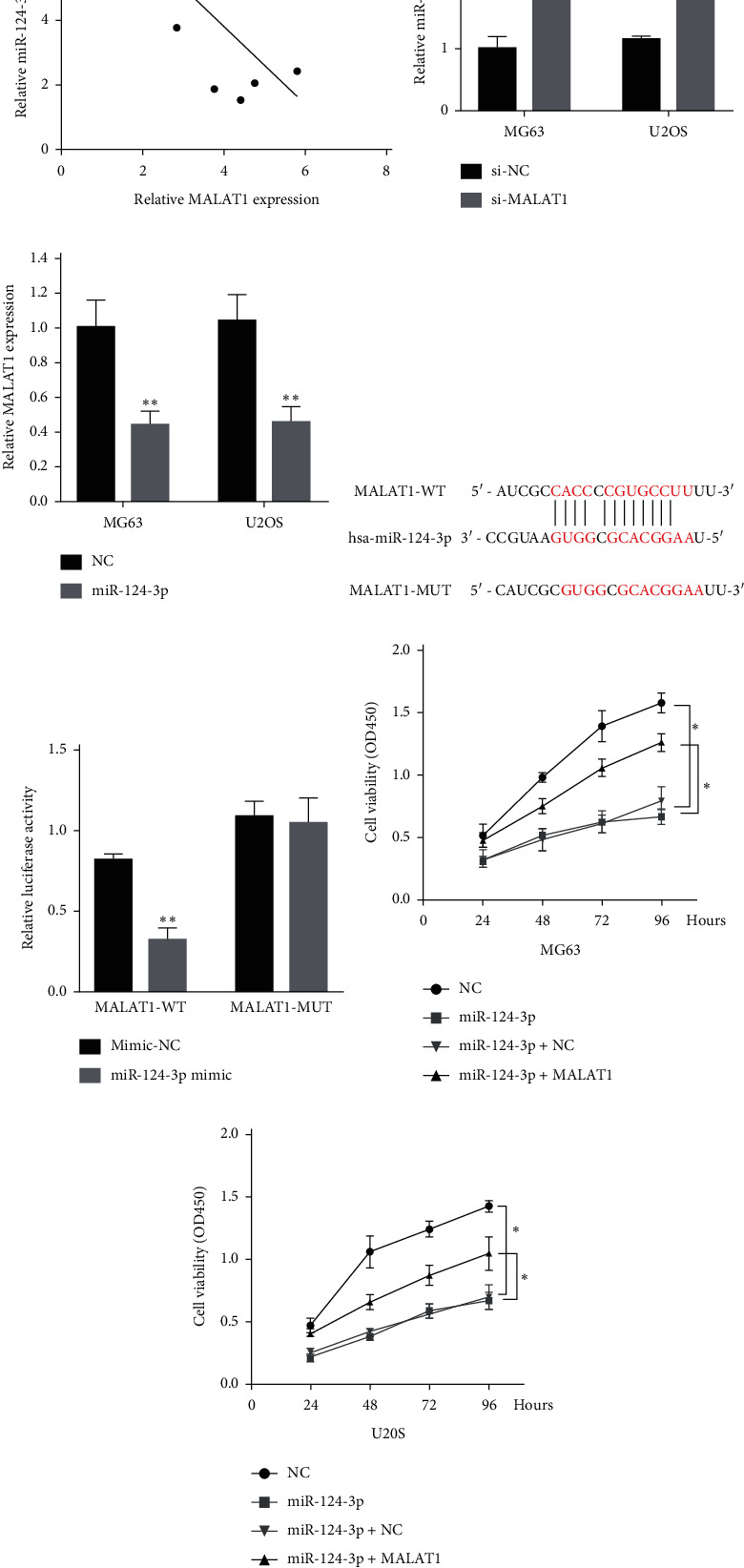
MALAT1 is associated with microRNA-124-3p (miR-124-3p) expression in OS tissues. (a) An inverse correlation between MALAT1 and miR-124-3p expression was observed in OS tissues. (b) Real-time PCR assay showed that knockdown of MALAT1 (si-MALAT1) caused upregulation of miR-124-3p in the MG63 and U2OS cell lines. (c) MALAT1 expression was decreased in response to miR-124-3p overexpression, compared with the miR-NC (NC) group. (d) Generation of MALAT1-WT and MALAT1-MUT containing luciferase reporter vectors by sequentially mutating the predicted miR-124-3p binding site in the MALAT1 3′-untranslated region (3′-UTR). (e) The MALAT1-WT/MALAT1-MUT vectors and miR-NC/miR-124-3p mimics were cotransfected into human embryonic kidney (HEK) 293T cells, respectively. miR-124-3p mimic significantly decreased the luciferase activities of MALAT1-WT compared with mimic-NC. Cotransfection with miR-124-3p mimic and MALAT1-MUT did not alter luciferase activities either. (f, g) MG63 and U2OS cells were transfected with NC, miR-124-3p, miR-124-3p + NC, or miR-124-3p + MALAT1. Cell viability was determined by CCK-8 assay in transfected MG63 and U2OS cells at 24, 48, 72, and 96 h. Results showed that MALAT1 overturns the miR-124-3p induced inhibitory effect on proliferation of OS cells. Data are presented as mean ± SD of three independent experiments. ^*∗*^*p* < 0.05 and ^*∗∗*^*p* < 0.01.

Accumulating evidence has suggested that lncRNAs could function as miRNA sponges and inhibit miRNAs activity. Does MALAT1 also regulate miRNAs in the form of sponge molecule in OS? First, we searched for miRNAs with complementary base pairing with MALAT1 using the online software Starbase v2.0, and a complementary binding site between miR-124-3p and the 3′-UTR of MALAT1 (Figure 3(d)) was identified. Next, we concentrated on miR-124-3p, a tumor suppressor involved in cancer cell proliferation and migration.

We found a negative linear relationship between the expression of miR-124-3p and MALAT1 in OS tissue (Figure 3(a)). The qRT-PCR assay showed that miR-124-3p expression was increased in the si-MALAT1 group when compared with the si-NC group (Figure 3(b)), while MALAT1 expression was decreased in the miR-124-3p overexpression group when compared with the negative control (NC) group (Figure 3(c)). Together, the above data suggested that expression of miR-124-3p is negatively correlated with expression of MALAT1 in OS cells.

We explored the targeted binding relationship between miR-124-3p and MALAT1 using dual luciferase assay in further experiments. We cloned the predicted miR-124-3p binding site of MALAT1 (MALAT1-WT) and a mutated binding site (MALAT1-MUT) into a luciferase reporter plasmid. Luciferase activity was assayed 24 h after transient cotransfection. The results showed that miR-124-3p mimic significantly decreased the luciferase activities of MALAT1-WT compared with mimic-NC. Cotransfection with miR-124-3p mimic and MALAT1-MUT did not alter luciferase activities either (Figure 3(e)). Together, these data suggested that miR-124-3p could directly bind to MALAT1 and decrease its expression.

To confirm the biological function of this targeted binding relationship, we observed the cell viability of MG63 and U2OS cells transfected with NC, miR-124-3p, miR-124-3p + NC, or miR-124-3p + MALAT1. CCK-8 results showed that miR-124-3p overexpression significantly inhibited cell viability in MG63 and U2OS cells when compared with the NC transfected group, whereas MALAT1 alleviated the inhibitory effect (Figures 3(f) and 3(g)). Collectively, the results indicated that targeted binding relationship between miR-124-3p and MALAT1 might present functional regulatory effect except for expression alterations.

### 3.3. Identifying the Regulatory Relationship between MALAT1, miR-124-3p, and SphK1

**Figure 4:**
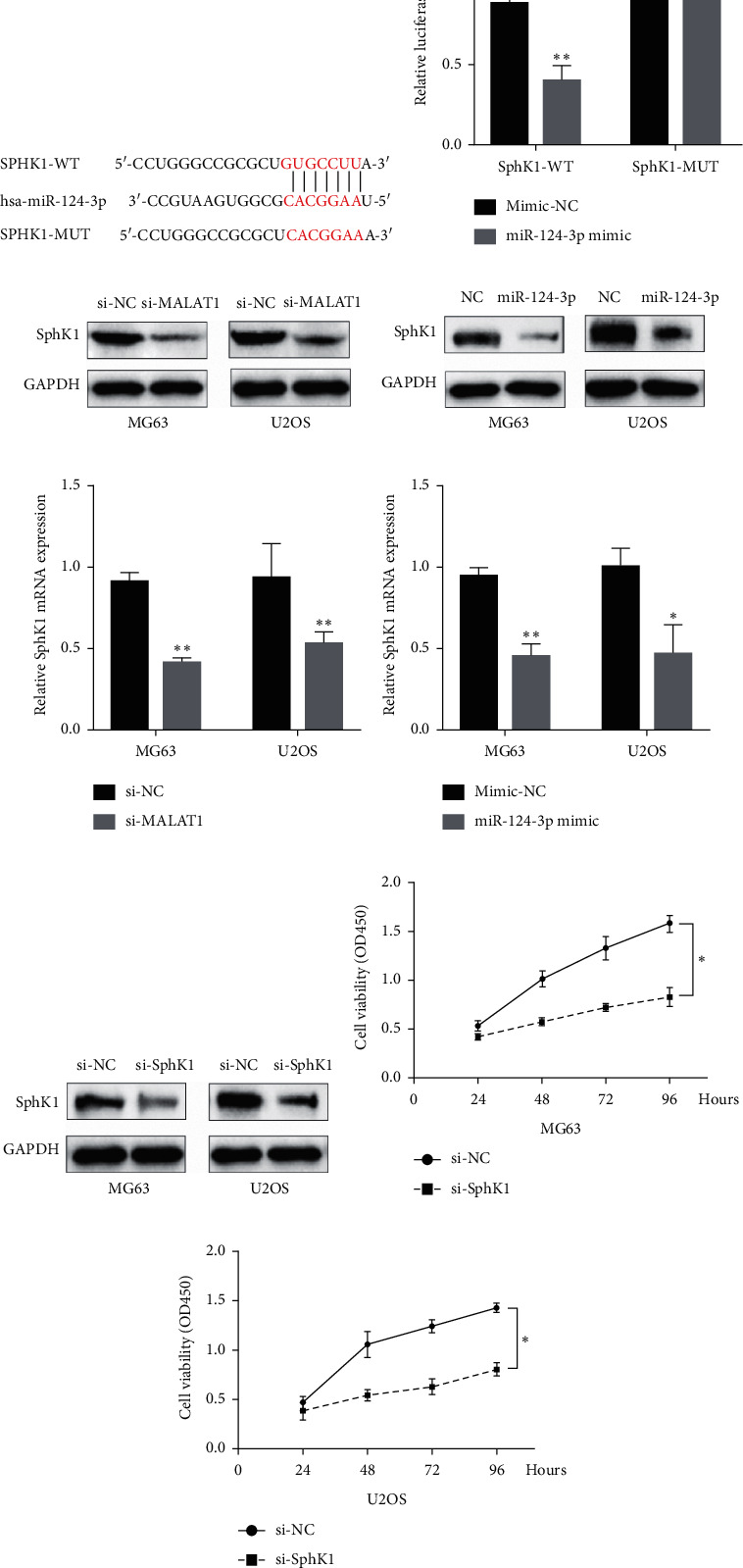
Identifying the regulatory relationship between MALAT1, miR-124-3p, and SphK1. (a) The putative binding sequences of miR-124-3p and 3′-UTR of SPHK1. (b) The SphK1-WT/SphK1-MUT vectors and miR-NC/miR-124-3p mimics were cotransfected into 293T cells, respectively. miR-124-3p mimic significantly decreased the luciferase activities of SphK1-WT compared with mimic-NC. Cotransfection with miR-124-3p mimic and SphK1-MUT did not alter luciferase activities either. (c, d) Western blot assay showed that the expression of SphK1 was downregulated by knockdown of MALAT1 and miR-124-3p overexpression in both MG63 and U2OS cells. (e, f) Real-time PCR assay also showed similar results in both MG63 and U2OS cell lines. (g) SphK1 knockdown was achieved by si-SphK1 as demonstrated by Western blot assay. (h, i) CCK-8 assay results showed that SphK1 inhibition by si-SphK1 reduced the proliferation of OS cells. Data are presented as mean ± SD of three independent experiments. ^*∗*^*p* < 0.05 and ^*∗∗*^*p* < 0.01.

Further analysis was conducted with a focus on the relationship between miR-14-3p and Sphk1. First, we predicted the binding sites between miR-124-3p and SphK1 using online prediction software TargetScan (Figure 4(a)). Then, we validated the targeted binding relationship between miR-124-3p and SphK1 using dual luciferase assay. We cloned the predicted miR-124-3p binding site of SphK1 (SphK1-WT) and a mutated binding site (SphK1-MUT) into a luciferase reporter plasmid. Luciferase activity was assayed 24 h after transient cotransfection. The results showed that miR-124-3p mimic significantly decreased the luciferase activities of SphK1-WT compared with mimic-NC. Cotransfection with miR-124-3p mimic and SphK1-MUT did not alter luciferase activities either (Figure 4(b)). Together, these data suggested that miR-124-3p could directly bind to SphK1 and decrease its expression.

To identify the regulatory relationship between MALAT1, miR-124-3p, and SphK1, we first explored the effect of miR-124-3p and MALAT1 on Sphk1 mRNA/protein expression in human OS cells. Sphk1 was downregulated by the knockdown of MALAT1 and overexpression of miR-124-3p, as demonstrated by Western blot (Figures 4(c) and 4(d)). Similar results were also observed in mRNA levels (Figures 4(e) and 4(f)). We next evaluated the effects of SphK1 on OS cells. Western blot results showed that si-SphK1 obviously decreased the SphK1 expression in MG63 and U2OS cells when compared with the si-NC group (Figure 4(g)). CCK-8 assays revealed that OS cells viability was decreased in response to SphK1 inhibition by si-SphK1 (Figures 4(h) and 4(i)). Together, these results suggested that SphK1 promotes proliferation in OS cells, and MALAT1 may interact with miR-124-3p to modulate OS progression by targeting SphK1.

## 4. Discussion

In recent years, numerous studies demonstrated that lncRNAs play a pivotal role in cancer development and progression, including breast cancer [11], gallbladder cancer [12], prostate cancer [13], and other malignancies, as well as OS [14]. Considering that biological behaviors of malignancies can be regulated by lncRNAs, they may be a potential therapeutic target in patients with malignant tumor.

lncRNA MALAT1 expression has been demonstrated increased in human OS, and it was shown to regulate the proliferation, invasion, and metastasis of OS cells via several signaling pathways [3, 4, 15, 16]. Moreover, lncRNA MALAT1 was shown to be an independent prognostic factor in OS [17]. In the present study, we demonstrated that lncRNA MALAT1 was markedly upregulated in OS tissues and cell lines when compared with adjacent healthy tissues and normal cell lines. We also found that knockdown of MALAT1 decreased proliferation and migration in OS cells. These results were concordant with the findings of previous studies, and it suggested MALAT1 could serve as a potential target for OS treatment.

As a member of miR-124 family, miR-124-3p could inhibit cell proliferation, and dysregulation of miR-124-3p has been demonstrated to be involved in tumorigenesis and progression in multiple tumor types, including breast cancer [7], bladder cancer [18], non-small cell lung cancer [19], and prostate cancer [20], as well as OS. Huang demonstrated that miR-124-3p was downregulated in OS, functioning as a tumor suppressor by attenuating OS cell proliferation and invasion. Moreover, miR-124-3p was associated with the adverse clinical and pathological features observed in OS [21]. Our previous study also found that miR-124-3p could function as a tumor suppressor in OS by targeting ROCK1 [22]. However, the underlying mechanism of miR-124-3p inhibiting OS remained unclear, requiring further study and exploration.

To explore the correlation between MALAT1 and miR-124-3p, we performed bioinformatics analysis, and the result demonstrated a putative binding site in MALAT1 for miR-124-3p. Next, a luciferase assay indicated that miR-124-3p indeed could bind to MALAT1 directly by the putative miRNA response element. In addition, MALAT1 knockdown led to an elevated miR-320b expression, while miR-124-3p overexpression suppressed MALAT1 expression. These results indicated the negative regulatory relationship between MALAT1 and miR-124-3p. Further experimental results revealed that MALAT1 could alleviate the proliferation inhibition mediated by miR-124-3p, suggesting negative regulation of miR-124-3p by the MALAT1 could regulate biological behavior in OS cells.

The sphingosine kinase 1 (SphK1) is a key regulator of the balance between pro-death sphingosine and ceramide and pro-survival sphingosine-1-phosphate (S1P). The role of SphK1 in the regulation of cell cycle progression through the G1/S phase has been well documented. As an oncogenic kinase, it exhibits high expression in many types of tumors, including breast cancer, colon cancer, lung cancer, ovarian cancer, gastric cancer, uterine cancer, renal cancer [23, 24], and acute leukemia [25], as well as OS [26]. Patients with SphK1 overexpression are often with poor prognosis [27]. Previous studies showed that the expression of SphK1 was significantly increased in OS tissues, and SphK1 proved to be a critical oncogene of OS, and it could promote growth of OS and endorsed its resistance against chemotherapeutic drugs [26]. Further study suggested that SphK1 participated in the development of doxorubicin resistance and contributed to glycolysis in OS cells by regulating HIF-1*α* expression [28]. In the present study, the CCK-8 assay showed that a downregulated expression of SphK1 reduced the proliferation of OS cells, which in turn proved that SphK1 promoted the proliferation of OS cells.

However, the relationship between SphK1, MALAT1, and miR-124-3p in OS is currently unclear. On the basis of proving the negative-regulation relationship between MALAT1 and miR-124-3p, we further identified that SphK1 was a potential target of miR-124-3p using a bioinformatics tool. Luciferase reporter assay and regulatory analysis in this study showed that miR-124-3p downregulated the expression level of SphK1. We also observed that overexpression of miR-124-3p or knockdown of MALAT1 both led to a significantly reduced SphK1 expression.

In recent years, a new RNA regulation mechanism—competing endogenous RNA (ceRNA)—has been proposed [29]. In this mechanism, RNAs can compete with each other miRNAs to bind with miRNAs, thereby regulating downstream RNAs, achieving posttranscriptional regulation, and participating in the regulation of biological behaviors of OS. For example, our previous study suggested that lncRNA HOXA11-AS acts as an endogenous sponge by directly binding miR-124-3p and decreasing the expression of miR-124-3p and then exerts an oncogene function in OS [22].

In the present study, we found that the expression of SphK1 was significantly downregulated after MALAT1 knockout and miR-124-3p overexpression. Combined with the negative regulatory relationship between MALAT1 and miR-124-3p, we believed that SphK1 expression is positively correlated with MALAT1 expression and negatively correlated with miR-124-3p expression.

Together, we speculated that MALAT1 acted as an endogenous sponge by directly binding to miR-124-3p and consequently decreasing the expression of miR-124-3p. Also, we found that MALAT1 may regulate OS progression by affecting miR-124-3p targeting SphK1 expression, indicating that MALAT1 functioned as a ceRNA to regulate SphK1 expression by sponging miR-124-3p in OS.

## 5. Conclusions

Overall, the present study demonstrated that MALAT1 overexpression facilitates cell proliferation and migration in OS. More importantly, our data revealed a novel MALAT1/miR-124-3p/SphK1 regulatory pathway in OS cells. Among them, MALAT1 could act as a competing endogenous RNA to bind miR-124-3p, then potentially promoting OS progression via targeting SphK1.

## Figures and Tables

**Table 1:** Primer sequence of PCR.

Gene name		5′-3′ sequence	Size (bp)
MALAT1	Forward	ACTGTAATGCTGGGTGGGAA	168
	Reverse	CATTGGAGATCAGCTTCCGC	

SphK1	Forward	TGACCAACTGCACGCTATTG	159
	Reverse	CCAGACGCCGATACTTCTCA	

GAPDH	Forward	TCAAGAAGGTGGTGAAGCAGG	115
	Reverse	TCAAAGGTGGAGGAGTGGGT	

miR-124-3p	Stemloop	GTCGTATCCAGTGCAGGGTCCGAGGTATTCGCACTGGATACGACTTGGCATT	
	F-PCR	TGCGCTAAGGCACGCGGTGAAT	

U6	Stemloop	GAATTTGCGTGTCATCCTTG	
	Forward	GCTTCGGCAGCACATATACTAAAAT	
	Reverse	CGCTTCACGAATTTGCGTGTCAT	

**Table 2:** Correlation between MALAT1 expression and clinical pathologic parameters of OS.

Clinicopathological features	Group	MALAT1 expression		*p* value
		Low	High	
Gender	Male	4	6	1.000
	Female	5	6	

Age (years)	<20	4	7	0.670
	≥20	5	5	

Anatomic location	Tibia/femur	7	8	0.659
	Elsewhere	2	4	

Clinical stage	I/IIA	6	2	0.032
	IIB/III	3	10	

Distant metastasis	Yes	2	11	0.002
	No	7	1	

## Data Availability

Data and materials would be made available upon request.

## Abbreviations

OS: Osteosarcoma
MALAT1: Metastasis associated lung adenocarcinoma transcript 1
LncRNA: Long noncoding RNA
SphK1: Sphingosine kinase 1.
